# Cancer Metabolism: New Validated Targets for Drug Discovery

**DOI:** 10.18632/oncotarget.1182

**Published:** 2013-07-22

**Authors:** Federica Sotgia, Ubaldo E. Martinez-Outschoorn, Michael P. Lisanti

**Affiliations:** ^1^ Manchester Breast Centre & Breakthrough Breast Cancer Research Unit, Faculty Institute of Cancer Sciences, University of Manchester, UK; ^2^ Kimmel Cancer Center, Thomas Jefferson University, Philadelphia, PA

**Keywords:** cancer metabolism, therapeutic targets, drug discovery, oncogenes, tumor suppressors, oxidative stress, glycolysis, cancer associated fibroblast, tumor microenvironment, metabolic symbiosis, anti-angiogenic therapy

## Abstract

Recent studies in cancer metabolism directly implicate catabolic fibroblasts as a new rich source of i) energy and ii) biomass, for the growth and survival of anabolic cancer cells. Conversely, anabolic cancer cells upregulate oxidative mitochondrial metabolism, to take advantage of the abundant fibroblast fuel supply. This simple model of “metabolic-symbiosis” has now been independently validated in several different types of human cancers, including breast, ovarian, and prostate tumors. Biomarkers of metabolic-symbiosis are excellent predictors of tumor recurrence, metastasis, and drug resistance, as well as poor patient survival. New pre-clinical models of metabolic-symbiosis have been generated and they genetically validate that catabolic fibroblasts promote tumor growth and metastasis. Over 30 different stable lines of catabolic fibroblasts and >10 different lines of anabolic cancer cells have been created and are well-characterized. For example, catabolic fibroblasts harboring ATG16L1 increase tumor cell metastasis by >11.5-fold, despite the fact that genetically identical cancer cells were used. Taken together, these studies provide >40 novel validated targets, for new drug discovery and anti-cancer therapy. Since anabolic cancer cells amplify their capacity for oxidative mitochondrial metabolism, we should consider therapeutically targeting mitochondrial biogenesis and OXPHOS in epithelial cancer cells. As metabolic-symbiosis promotes drug-resistance and may represent the escape mechanism during anti-angiogenic therapy, new drugs targeting metabolic-symbiosis may also be effective in cancer patients with recurrent and advanced metastatic disease.

Metabolic-symbiosis represents a paradigm shift in cell biology and cancer metabolism [1-20]. In this simple metabolic model, catabolic fibroblasts fuel the growth of adjacent anabolic cancer cells, via energy transfer (Figure 1) [2-4, 7, 12, 13, 15, 17, 19-53]. Catabolic stromal cells produce high-energy mitochondrial “biofuels”, such as L-lactate, ketone bodies, glutamine, other amino acids, and free-fatty acids, for cancer cells to use as substrates for OXPHOS and as biomass. [38, 40].

**Figure 1:**
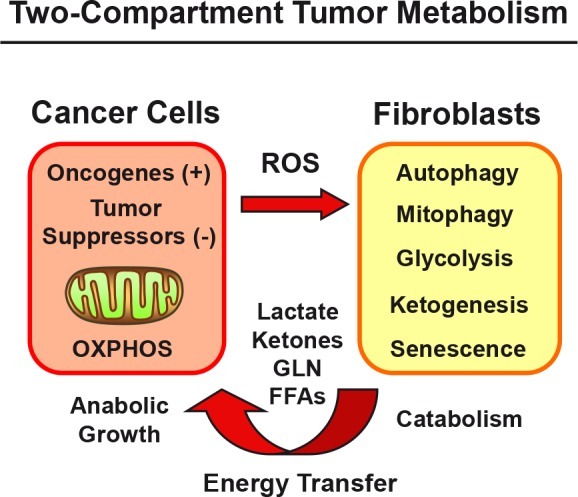
Metabolic-Symbiosis in Human Cancer(s): New Therapeutic Targets Two-Compartment Tumor Metabolism: Schematic Diagram. Catabolic stromal fibroblasts produce high-energy mitochondrial fuels, for cancer cells to use as substrates for OXPHOS and as biomass. Oncogenes (gain-of-function) and tumor suppressors (loss-of-function) both induce catabolism in adjacent fibroblasts, via ROS production (hydrogen peroxide) and the onset of oxidative stress.

Catabolic fibroblasts also show a pro-inflammatory phenotype, due to oxidative stress and NFkB activation, which leads to cytokine production. This, in turn, attracts and serves to activate inflammatory cells (macrophages and neutrophils), which produce more ROS and hydrogen peroxide species. These findings link inflammation directly with energy transfer to anabolic cancer cells [2, 41, 42, 54, 55], explaining how inflammation energetically promotes tumor initiation and cancer progression.

To stringently test the validity of these energy transfer mechanism(s), stable cell lines of constitutively catabolic fibroblasts were generated by genetically increasing glycolysis, ketogenesis, autophagy, mitophagy, oxidative stress, and/or senescence. This was accomplished by the recombinant over-expression or knock-down of key metabolic target genes in hTERT-immortalized fibroblasts. Similar results were obtained by the genetic manipulation of either growth factors or extracellular matrix proteins, indicating that these “signaling networks” also converge on catabolic metabolism in stromal fibroblasts.

These results are summarized in Table 1, which lists nearly 30 catabolic fibroblast cell lines that have been generated, to date [1-20]. Remarkably, these catabolic fibroblasts [56] effectively promoted tumor growth and/or metastasis, in pre-clinical animal models (xenografts in nude mice) [1-20]. Similar results have also been obtained by using a syngeneic orthotopic animal model, employing the mammary fat pads of Cav-1 (−/−) null mice, as the catabolic host microenvironment for tumor growth [57].

**Table 1:** New Validated Targets in Cancer Metabolism

**A. Catabolic Cancer-Associated Fibroblasts**	**Ref.**
Glycolysis/Ketogenesis	
CAV1, HIF1A, PKM1, PKM2, CA9, HMGCS2, BDH1, MCT4	[1-6]
Autophagy/Mitophagy/Inflammation	
ATG16L1, CTSB, BNIP3, BNIP3L, BECLIN1, NFkB, DRAM, LKB1	[2, 7, 8]
Mitochondrial Dysfunction	
TFAM, MFF, UCP1	[9-11]
Senescence and Cell Cycle Arrest	
p16-INK4A, p19-ARF, p21-CIP1/WAF1	[12]
Growth Factors/Extracelluar Matrix Proteins	
CTGF, TGF-beta1/2/3, TGF-beta type I receptor kinase, Migration Stimulating Factor (MSF), PAI1, PAI2, PPAR-gamma receptor	[13-17]
**B. Anabolic Epithelial Cancer Cells**	
Mitochondrial Hyper-function	
PGC1A, PGC1B, MitoNEET, POLRMT, GOLPH3, HIF2A	[8, 18, 19]
Lactate and Ketone Metabolism	
ACAT1, ACAT2, OXCT1, OXCT2, MCT1	[4, 20]

A. Catabolic fibroblasts were generated by recombinant over-expression or knock-down of key metabolic target genes in hTERT-immortalized fibroblasts. Similarly, catabolic fibroblasts were also obtained by the genetic manipulation of either growth factors or extracellular matrix proteins. Nearly 30 catabolic fibroblast cell lines that have been generated, are listed. Remarkably, these catabolic fibroblasts promoted tumor growth and/or metastasis, in pre-clinical animal models (xenografts in nude mice).

B. Over-expression of genes that drive mitochondrial biogenesis or augment ketone metabolism in MDA-MB-231 epithelial cancer cells, also effectively promoted tumor growth, and induced autophagy-resistance.

Conversely, over-expression of metabolic genes that drive increased mitochondrial biogenesis or OXPHOS in epithelial cancer cells, also effectively promoted tumor growth, and induced autophagy-resistance (Table 1) [4, 8, 18-20].

As metabolic-symbiosis may represent the underlying basis of drug-resistance [31, 32], and/or the escape mechanism [35, 43, 44, 47, 48] during anti-angiogenic therapy [53], new drugs that target metabolic-symbiosis may prove to be effective in patients with recurrent cancers and even for the treatment of advanced metastatic disease [25-27, 35, 43].

The existence of metabolic-symbiosis (a.k.a., two-compartment tumor metabolism) has also been directly validated in human breast cancer tissue sections, by employing mitochondrial activity staining *in situ*. Using this approach, it is clear that oxidative, mitochondrial-rich cancer cell nests, are physically surrounded by glycolytic, mitochondrial-poor stromal fibroblasts (Figure 2) [58]. Virtually identical results were also obtained with metabolic protein biomarkers in primary breast tumors and secondary lymph-node metastases (Figure 3), reflecting a common organizing principle, with the juxtaposition of oxidative and glycolytic energetic compartments [52, 59]. As such, tumor architecture also “mirrors” these energy-based tumor-stromal interactions.

**Figure 2:**
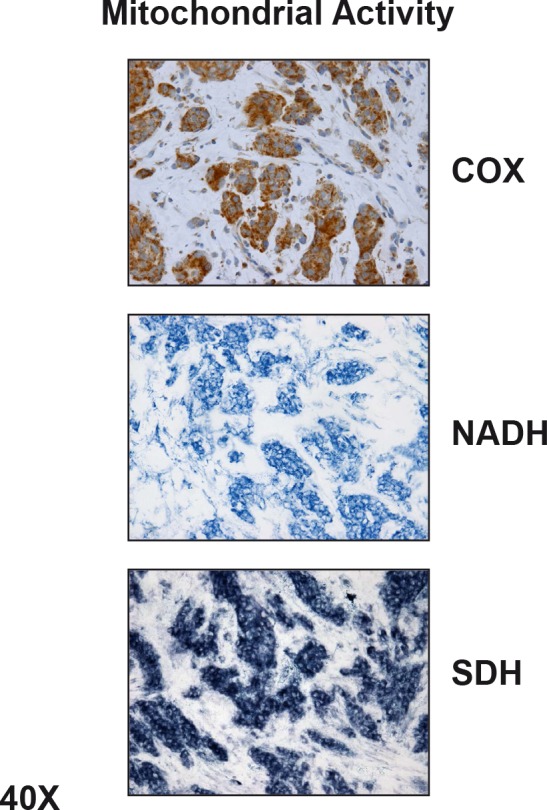
Mitochondrial Activity Staining in Fresh Frozen Human Breast Cancer Tumor Tissue Sections Note that epithelial cancer cell “nests” amplify their mitochondrial metabolism. In contrast, surrounding stromal fibroblasts show little or no functional mitochondrial staining, indicating that they show a shift towards glycolysis. COX, NADH, and SBH represent functional activity staining for mitochondrial complex IV, I, and III, respectively. Reproduced, with permission, from [58].

**Figure 3:**
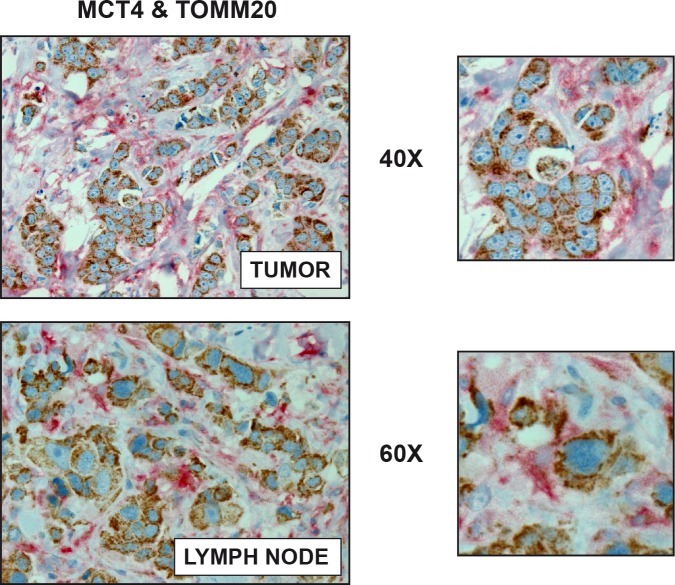
Visualizing Two-Compartment Tumor Metabolism, with Metabolic Marker Proteins: MCT4 and TOMM20 Human breast cancer samples (from primary tumors or lymph node metastases) were immuno-stained with antibodies directed against MCT4 (shown in RED) and TOMM20 (shown in BROWN). MCT4 is a marker of cellular stress, such as ROS production, glycolysis, and mitochondrial dysfunction, which reflects catabolic metabolism in cancer-associated fibroblasts. Conversely, TOMM20 is a marker of mitochondrial mass that has been shown to correlate with oxidative mitochondrial metabolism. Note that two distinct metabolic compartments (oxidative vs. glycolytic) co-exist, side-by-side, in human primary tumors. Virtually identical results were obtained with metastatic breast cancer lesions, within lymph node tissue. Insets are also shown at higher magnification. Reproduced, with permission, from [52].

Remarkably, new studies suggest that normal adjacent epithelial cells, and stromal adipocytes, can also serve as functional metabolic partners for anabolic cancer cells [22, 40, 56, 60]. Therefore, cancer cells may be able to use many different cell types, in addition to fibroblasts, as partners to engage in metabolic-symbiosis [52, 61].

Finally, oncogene-transformed epithelial cancer cells also show significant increases in mitochondrial mass, which is strictly dependent on oxidative stress [56]. Figure 4 shows that NAC treatment (N-acetyl-cysteine; an anti-oxidant) dramatically reduced mitochondrial staining, selectively in Ras-transformed cancer cells, but not in matched normal control epithelial cells. Thus, NAC selectively blocks mitochondrial biogenesis in Ras-transformed cells, illustrating how new drug discovery of more powerful anti-oxidants could be used therapeutically to “starve” cancer cells. Quantitation indicated that the mitochondrial marker TOMM20 was decreased by >5-fold during NAC-treatment [56]. As such, oxidative stress and ROS production may drive mitochondrial biogenesis in certain aggressive epithelial cancer cells.

**Figure 4:**
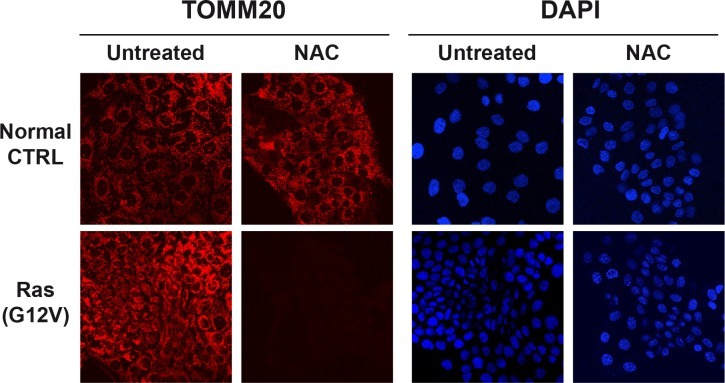
Anti-Oxidants Halt Mitochondrial Biogenesis Selectively in Cancer Cells, But Not in Non-transformed Epithelial Cells Epithelial cells (control versus H-Ras (G12V) transformed) were maintained (plus or minus NAC (10 mM)) and then subjected to immuno-staining with TOMM20, which is a well-established mitochondrial marker. Note that Ras-transformed cells, treated with NAC, show a significant decrease in mitochondrial mass. DAPI (blue nuclear staining) is also shown. Reproduced, with permission, from [56].

In summary, Table 1 lists >40 validated therapeutic target(s), related to metabolic-symbiosis, that could be exploited for new drug discovery. Thus, we should consider metabolic-symbiosis as a novel conceptual framework or platform to design more effective anti-cancer therapies.
